# Multivalent Carbonic Anhydrases Inhibitors †

**DOI:** 10.3390/ijms20215352

**Published:** 2019-10-28

**Authors:** Fabrizio Carta, Pascal Dumy, Claudiu T. Supuran, Jean-Yves Winum

**Affiliations:** 1 Department of Neurofarba, University of Florence, 50019 Sesto Fiorentino, Italy; claudiu.supuran@unifi.it; 2 Institut des Biomolécules Max Mousseron (IBMM), École nationale supérieure de chimie de Montpellier (ENSCM), Université de Montpellier, CEDEX 05, 34296 Montpellier, France; pascal.dumy@enscm.fr

**Keywords:** carbonic anhydrases, inhibitors, multivalency, multivalent, CA isoforms, multifunctionnal scaffold

## Abstract

Biomolecular recognition using a multivalent strategy has been successfully applied, this last decade on several biological targets, especially carbohydrate-processing enzymes, proteases, and phosphorylases. This strategy is based on the fact that multivalent interactions of several inhibitory binding units grafted on a presentation platform may enhance the binding affinity and selectivity. The zinc metalloenzymes carbonic anhydrases (CAs, EC 4.2.1.1) are considered as drug targets for several pathologies, and different inhibitors found clinical applications as diuretics, antiglaucoma agents, anticonvulsants, and anticancer agents/diagnostic tools. Their main drawback is related to the lack of isoform selectivity leading to serious side effects for all pathologies in which they are employed. Thus, the multivalent approach may open new opportunities in the drug design of innovative isoform-selective carbonic anhydrase inhibitors with biomedical applications.

## 1. Introduction

Human carbonic anhydrases (hCA, EC 4.2.1.1) are ubiquitous zinc enzymes of medical relevance that belong to the lyase family. These metalloproteins are efficient catalysts for the hydration of carbon dioxide to bicarbonate and protons playing crucial physiological/pathological roles in acid-base homeostasis, secretion of electrolytes, transport of ions, biosynthetic reactions, and tumorigenesis. X-ray crystallography data showed that zinc ion is situated at the bottom of a 15 Å deep active site cleft being coordinated by three histidine residues and a hydroxide ion. The catalytic mechanism takes place by the attack of HO^−^ on the CO_2_ molecule located in a neighboring hydrophobic pocket leading to the formation of bicarbonate coordinated to Zn ion. The bicarbonate is then displaced by a water molecule and released into the solution leading to the catalytically inactive acid form of the enzyme. The regeneration of the basic form is carried out by a proton transfer reaction from the active site to the environment with the assistance of histidine residues. There are 15 known isoforms in humans, and 12 of them are enzymatically active, and thus considered targets of carbonic anhydrase inhibitors (CAIs). By taking advantage of 40 years of research and with the help of structural studies, different drug design campaigns inspired new ideas and contributed significantly to the discovery of effective inhibitors with diuretics, antiglaucoma, anticancer, or antiobesity properties, or for the management of a variety of neurological disorders, including epilepsy and altitude disease [1].

Nevertheless, even if the potency of carbonic anhydrase inhibitors has been greatly improved in the last years, with inhibitors reaching inhibition constant in the femtomolar range, the selectivity issue remains important. Small molecules approach, featuring classic medicinal chemistry and rational drug design, is still relevant and, therefore, always the subject of intense research efforts [2].

Another interesting strategy that appeared recently to address the selectivity of CAIs against specific isoforms is the use of multivalent CA-directed pharmacologic agents. The idea behind this approach is that considering the thermodynamic modifications in binding upon going from the monovalent to the multivalent systems can be exploited to improve affinity but above all the selectivity, meaning that an inhibitor may become more effective and more selective in a cluster than alone. Moreover, the multivalency approach could be leveraged for changing low-affinity and weakly selective inhibitors into potent and selective inhibitors [3].

Multivalent nanoconstructs are generally prepared by the conjugation of inhibitors onto high or low valency preorganized (such as for example cyclopeptide) or disordered (such as dendrimers) multifunctional (bio)molecular chemical framework using different conjugation methodologies, especially click reactions [3].

Multimeric enzyme inhibition is not something particularly easy to implement. Attaching multiple inhibitors to a molecular platform does not necessarily lead to an effective multivalent system knowing that structural parameters such as topology, structure, and valency of the platform, length and rigidity of the ligation system, are among the many parameters to be refined in order to observe and improve multivalent effects.

This approach is thus of great interest for the medicinal chemist, in particular, for the design of enzyme inhibitors. It has been successfully used in glycosidase and glycosyltransferase inhibition [3,4,5,6]. Almost restricted to these family of carbohydrate-processing enzymes, few research works were reported on other enzyme families such as metalloproteases [7].

In the field of zinc metalloenzymes, pioneering work had been initiated in 2003 in the context of divalent CAIs exploiting the hydrophobic and the hydrophilic binding sites of hCA isoforms [8,9,10]. The interesting results obtained from these studies have contributed to the development of multimeric CAIs and our research groups were the first to report the application of this strategy on carbonic anhydrases using gold nanoparticles [11].

In this paper, we shall review the state of the art of the multivalent inhibition approach applied to carbonic anhydrases and its potential in both diagnostic and therapy.

## 2. Nanoparticles

Nanoparticles coated with CAIs revealed to be particularly effective in targeting selectively the hCA isoforms expressed extracellularly, such as the tumor-associated IX [11,12,13,14]. Since nanomaterial usually possesses the size and physicochemical features which do not allow them to bypass the cellular membranes, and thus to access into the intracellular lumen, they represent ideal platforms to address target selectivity and multivalent effect. Usually, the contact of nano-objects with the cellular membranes activates internalization processes by endocytosis, which still prevents xenobiotics from getting in contact with the cell machinery located within the cytosol.

### 2.1. Gold Nanoparticles and Nanorods

**Scheme 1:**
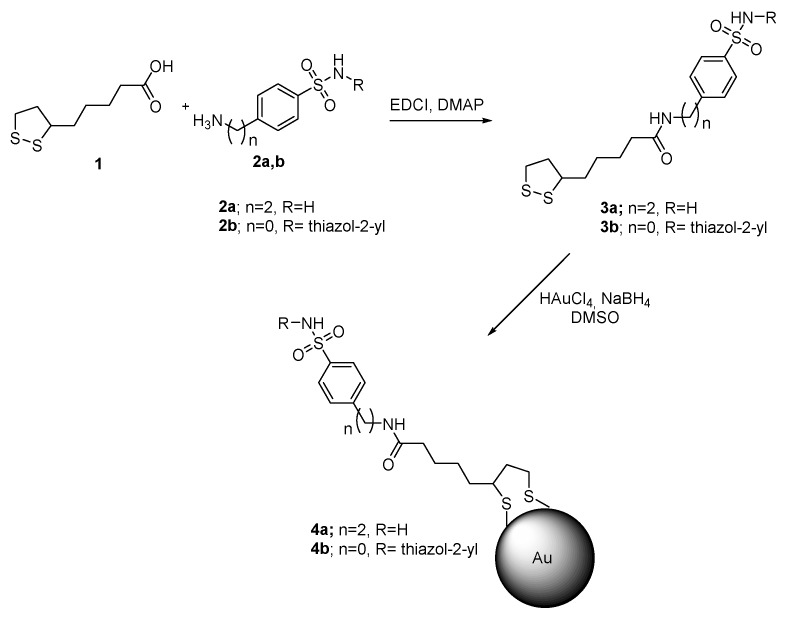
Synthesis of CAI coated gold nanoparticles [11].

Some of us firstly proved the use of gold nanoparticles fully coated with CAIs of the sulfonamide type, which was synthesized according to Scheme 1 reported below [11].

The synthetic procedure to obtain the desired organic intermediates consisted of ordinary amide reaction couplings of an appropriate amine on the (±) lipoic acid **1** moiety. The latter was chosen as for its 1,2-dithiolanic moiety, which ensures a tight binding to gold shells as well as acts as an optimal spacer, and thus, it prevents the sulfonamide moieties the be too close to each other once the nanoparticle surface is functionalized. The full coated shell was synthesized in one-pot reaction by reduction of acid chloroaurate with NaBH_4_ in the presence of the lipoic amides **3a** and **3b** previously reported.

The same strategy was successfully used to prepare both the CAI and the thiazolyl coated gold nanoparticles **4a** and **4b**, which were fully characterized by means of Transmission Electron Microscopy (TEM), Energy Dispersive X-ray analysis (EDX), and elemental analyses. All the data retrieved showed the nanoparticles being monodispersed, roughly spherical in shape and with an average size of 3.3 nm, which corresponds to 720–724 Au atoms. A calculated estimation indicated the number of ligand molecules per particle being of 144 and 135 for **4a** and **4b**, respectively. The synthetic procedures were reproducible as multiple batch preparations afforded coated nanoparticles with almost superimposed features [11].

**4a** and **4b** were tested in vitro for their ability in inhibiting the CA catalyzed CO_2_ hydration reaction by means of the stopped-flow technique and compared to the standard CAI acetazolamide (**AAZ**) (Table 1). The study focused on the physiologically relevant hCAs I, II, and IX isoforms.

The kinetic assay performed at 15 min incubation showed **3a** and **4a** being modest hCA I inhibitors (*K*_I_s of 214–581 nM), comparable to the reference **AAZ** (*K*_I_ 250 nM). In analogy, **3a** and **4a** showed high nanomolar *K*_I_ values against the hCA II (*K*_I_s of 230–451 nM). Predictably **3b** and **4b**, which are devoid of any CAI functionality, resulted ineffectively. Quite interestingly, **3a** and its corresponding coated nanoparticle **4a** were quite potent inhibitors of the tumor-associated hCA IX with *K*_I_ value of 41 and 32 nM, respectively, and thus comparable to the reference **AAZ** (*K*_I_ of 25 nM), whereas the remaining compounds were ineffective. The same experiments were also repeated after 2 h incubation (Table 1 numbers in brackets) and interestingly **4a** (but not its free ligand) showed enhanced inhibitory activity against all three isozymes (*K*_I_s of 128, 116 and 34 nM against hCA I, II, and IX, respectively). Overall, **4a** showed good selectivity in inhibiting the tumor-associated isoform in vitro. Since the kinetic assays were performed in solution on the hCA IX catalytic domain, such results may be rationalized only with the preferential binding of the CAI coated nanoparticle with specific amino acidic residues exposed within the enzymatic cleft. The penetrability of CAIs **3a**, **4a**, and **AAZ** through membranes was investigated by determining the erythrocyte expressed hCAs I and II inhibition. The measurements were performed up to 24 h time using millimolar concentrations of the CAIs. The results clearly showed **AAZ** and sulfonamide **3a** led to saturation of the two isozymes after 30–60 min, whereas **4a** was not able to determine any response, thus proving that the CAI coated nanoparticles were unable to penetrate through biological membranes.

As an extension of such an investigation, we set up a one-pot preparation method of aqueous dispersions of CAI-coated gold nanoparticles on the **4a** type, based on a modified citrate synthesis procedure, with the final intent to obtain highly dispersible and stable formulations [10]. All the obtained compounds were properly characterized for their physical-chemical features by means of Dynamic Light Scattering (DLS), Small Angle X-ray Scattering (SAXS), electrophoretic mobility, UV-visible spectroscopy, Inductively Coupled Plasma-Optical Emission Spectrometry (ICP-OES), and Thermogravimetric Analysis (TGA) experiments. The optimized procedure allowed to obtain high reproducibility CAI coated nanoparticles dispersed in an aqueous medium, which proved to be stable for months at room temperature (r.t.). The kinetic assay of such samples on the hCAs I, II, IX, and XII matched the results previously reported [11], and thus, giving further sustainment to the proof on the use of CAI coated gold nanoparticles as efficient and reliable systems to properly target hCAs for biomedical purposes [11,12].

**Scheme 2:**
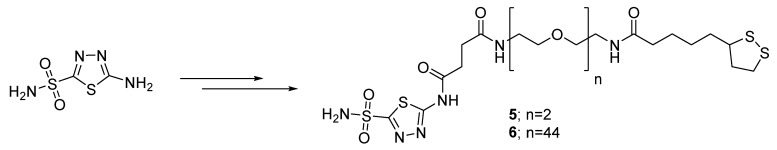
Synthesis of **AAZ**-based coatings **5** and **6** [13].

A quite advanced application on CAI-functionalized gold nanoparticles for the management of hypoxic tumors was recently reported by the Ilies group in 2018 [13]. On the basis of the author’s previous contributions [13], this work considered the use of the acetazolamide (**AAZ**) CAI intermediates **5** and **6** as coatings, and their synthesis was performed according to Scheme 2.

**Figure 1:**
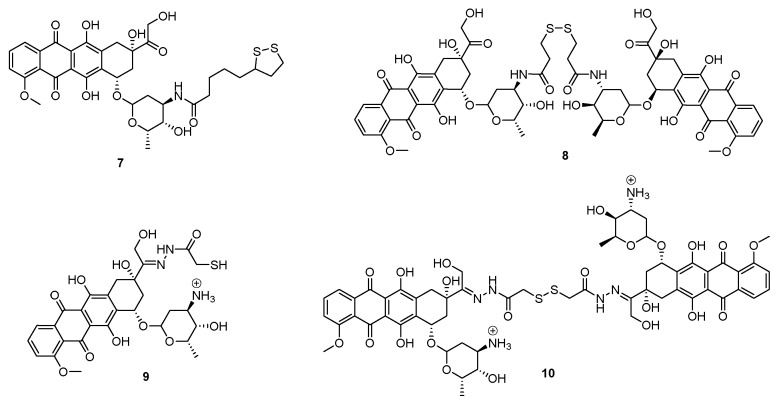
Structure of Doxorubicin conjugates **7**–**10** used for loading onto the CAI gold nanoparticles [13].

As previously discussed, also in this case, the authors did use the 1,2-dithiolane moiety of the (±)-lipoic acid as an anchoring group to the gold nanoparticles surface which was produced by the classical citrate method [13]. The materials obtained were thus characterized and properly assessed for their ability to interact with tumor cells. In particular, the in vitro tests carried out on colorectal HT-29 cell lines showed the nanoparticles bearing the CAI coating of type **6** were significantly internalized when hypoxic conditions were established. Such a result was rationalized as mediated by endocytosis uptake processes, which were triggered after binding of the CAI functionalized nanoparticles with the hypoxic expressed hCA IX. Different strategies to maximize the loading of doxorubicin (Dox) on the CAI functionalized nanoparticles were explored by using the precursors reported in Figure 1.

Among the various structures reported above, the conjugate **10** best maintained the appropriate physical-chemical features of the entire system. The in vitro tests on HT-29 cell lines clearly showed the high ability of such functionalized nanoparticles in killing them quite efficiently under hypoxia and thus when the hCA IX is overexpressed. Analogous results were obtained when HT-29 spheroids were used. Fluorescence spectroscopy proved that doxorubicin delivery by using the CAI coated nanoparticles resulted enhanced when compared to the standard administration in solution. Overall, such a work proofed the use of CAI coated nanoparticles as highly efficient and selective delivery systems of considerable cytotoxic payloads, and thus paving the way to introduce more effective pharmacological treatments of hypoxic tumors overexpressing the hCA IX.

### 2.2. Silica Nanoparticles

**Scheme 3:**
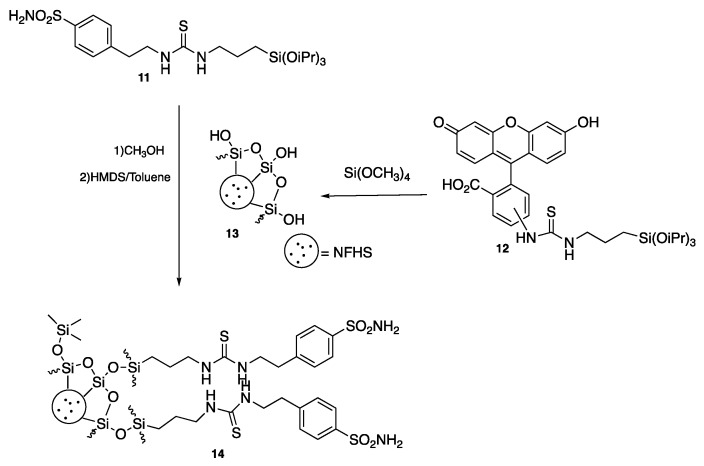
Synthesis of NFHS **14** coated with sulfonamides.

In the continuity of our work on nanoparticle-type platforms, we reported in 2015, the design and the synthesis of multivalent nanometer-sized fluorescent hybrid silica (NFHS) decorated with sulfonamide carbonic anhydrase inhibitors. [14] They were prepared by grafting silylated inhibitor ligand **11**, on the fluorescent silica nanoparticle **13** previously obtained by co-hydrolysis and polycondensation of the fluorescent silylated precursor **12** and tetramethoxysilane under basic catalysis (Scheme 3) [14]. NFHS **14** with a diameter of about (3.5 ± 0.8) nm were characterized as non-porous, and the evaluation of the number of sulfonamide ligands was estimated between 12 and 14.

Inhibition study realized with a stopped-flow CO_2_ hydration assay method against cytosolic isoforms hCA I, hCA II as well as the two tumor-associated isoforms hCA IX and hCA XII showed a low nanomolar activity of NHFS **14** with inhibitory activity ranging from 0.67 to 6.2 nM against all tested isozymes. Comparison of the inhibitory activity of the multivalent NHFS **14** vs. the monovalent inhibitor, revealed an increased inhibitory potency for the multivalent system respectively of 32 times for hCA I, 72 times for hCA II, 14 times for hCA IX and 5 times for hCA XII. Multivalent effects were detected only with hCA I and hCA II with a relative potency normalized to the sulfonamide units (*rp*/*n*) being equal respectively to 2.7 and 6. No multivalent effect was observed for hCA IX and hCA XII. The difference of the effect was hypothesized to come from steric hindrance effects as hCA I and II are monomeric and hCA IX and XII dimeric enzymes. This may also suggest that multivalent binding occurs through enzyme clustering. [14]

Currently, other silica nanoobjects are investigated as potential multivalent systems with potential applications in the field of carbonic anhydrase inhibition. [15]

### 2.3. Gold Nanorods

Nanorods did become particularly attractive within the nanomaterial-based compounds for biomedical applications, as they possess specific optical and thermal features that make them proper innovative tools. Nanorods are perfectly suited for Near-InfraRed light (NIR) absorption and conversion of its energy into thermal radiation with the generation of very minimal structural defects to the material after its exposure to multiple cycles [16,17,18]. Such an effect referred as Surface Plasmonic Resonance (SPR), is well known in nanodevices [16,17,18]. Nanorods have the advantage to possess their SPR-inducing frequency within the NIR spectral window (i.e., 750–1200 nm), and that allows them to make use of the deep penetration ability and low interference with biological tissues of this radiation [16,19]. In this contest, we proofed that CAI coated nanorods selectively target hypoxic tumor cells and determine death after exposure to NIR [16].

**Figure 2:**
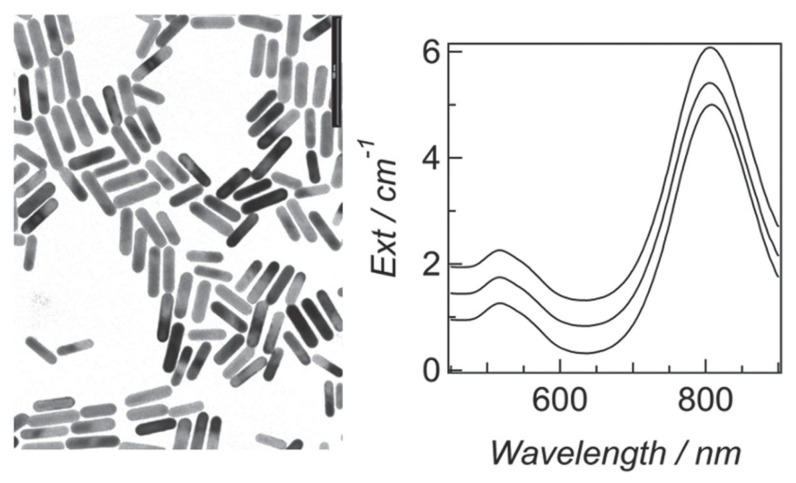
Representative TEM of synthesized gold nanorods. And optical extinction spectra of cetrimonium-terminated **15**, CAI- and 2-methoxyethylamine conjugated particles **16** and **17** in aqueous suspensions, respectively, from bottom to top [21].

The synthesis of gold nanorods **15** was achieved by means of autocatalytic reduction of chloroauric acid with ascorbic acid in the presence of cetrimonium bromide, silver nitrate, and ultra-small gold nanospheres according to the procedure described in the literature [20]. Then the gold nanorods were subjected to PEGylation with commercially available heterobifunctional PEG strands composed of 90% α-mercapto-ω-methoxy and 10% α-mercapto-ω-carboxy PEG (MW ≈ 5000 g/mol). The PEGylated nanorods obtained were further functionalized by means of standard *N*-hydroxysuccinimide/1-ethyl-3-(3-dimethylaminopropyl)carbodiimide (NHS/EDCI) catalyzed amidation protocols to afford the CAI ethylaminobenzenesulfonamide coated nanorods **16** and the 2-methoxyethylamine derivative nanorods **17** as a negative control. The obtained gold shells 15, as well as the coated nanorod batches **16** and **17**, were properly characterized for their physical and optical properties by mean of Transmission Electron Micrograph (TEM) and optical extinction spectra (Figure 2).

As reported in Figure 2, the optical extinction characterization spectra of all nanorods were almost superimposable as only small variations were detected. This implies that the particles did not aggregate upon the modification and therefore were able to retain their optical features [21].

**Figure 3:**
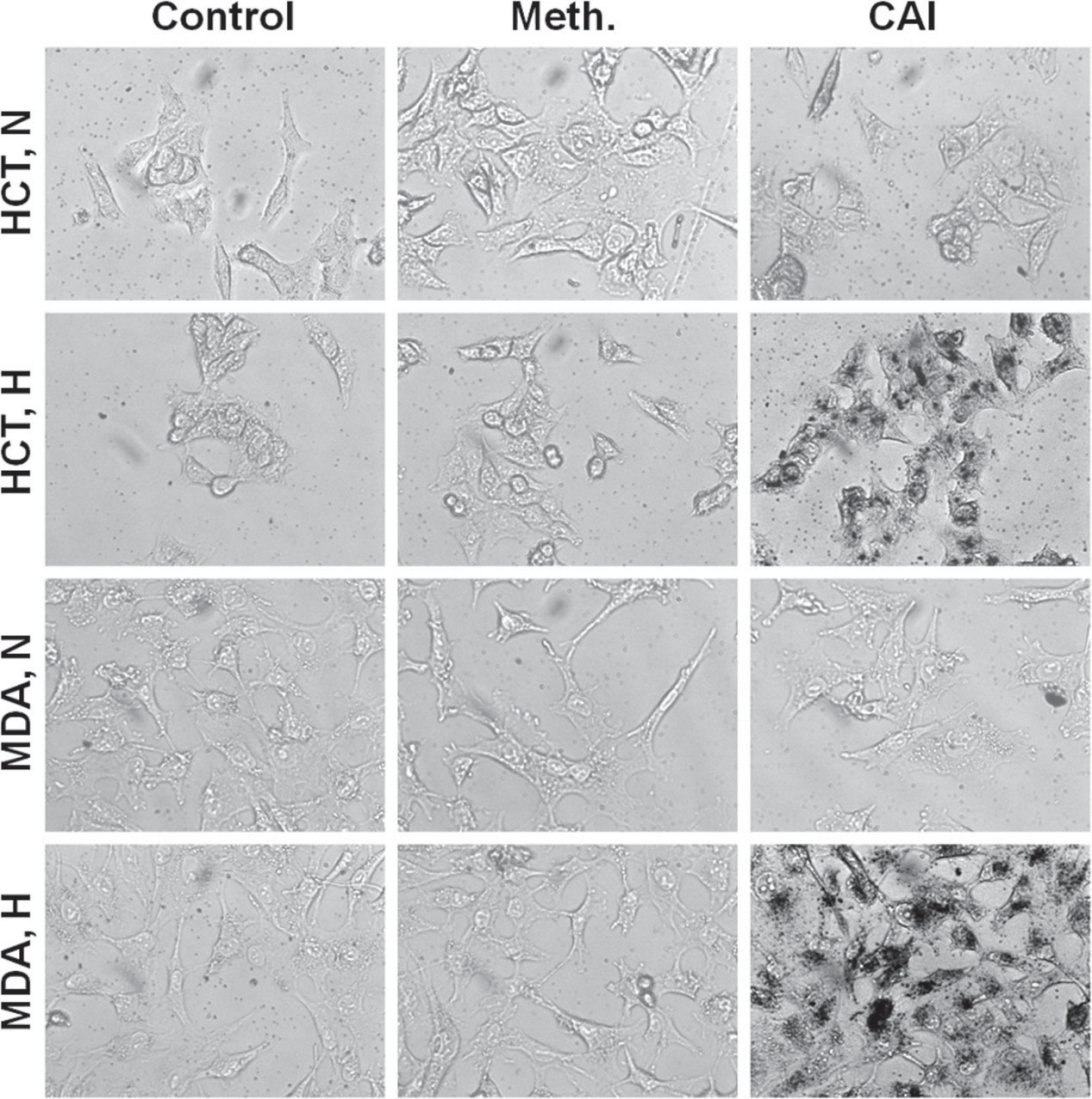
350 μm × 250 μm Optical micrographs of HCT or MDA cells in normoxia (N) and hypoxia (H), treated without particles (Control) or with 100 μM of CAI-terminated particles **16** or 2-methoxyethylamine- (Meth.) **17** and finally stained with silver [21].

The most relevant aspect of the study for the purposes of this review is the assessment of the biological features of the CAI coated nanorods, which showed no cytotoxicity on human colorectal carcinoma HCT116 (HCT) and human mammary adenocarcinoma MDA-MB-231 (MDR) cells after exposure to 24 h. Both HCT and MDR cell lines under normoxic and hypoxic conditions were incubated with CAI- and 2-methoxyethylamine capped nanorods **16** and **17** and subjected to silver staining. The corresponding optical micrographs clearly visualized the preferential accumulation of gold nanorods for both HTC and MDR cell cultures incubated under hypoxia and treated with CAI-functionalized nanorods **16** (Figure 3). Such results were also confirmed with quantitative analyses, which accounted for the HTC cell lines retaining remarkable amounts of gold nanoparticles with respect to the MDR series (data not reported here). Overall such results were in agreement with our hypothesis on the strong interactions occurring between the sulfonamides end-capping of the nanorods with the membrane expressed and hypoxia-induced hCA IX [21].

**Figure 4:**
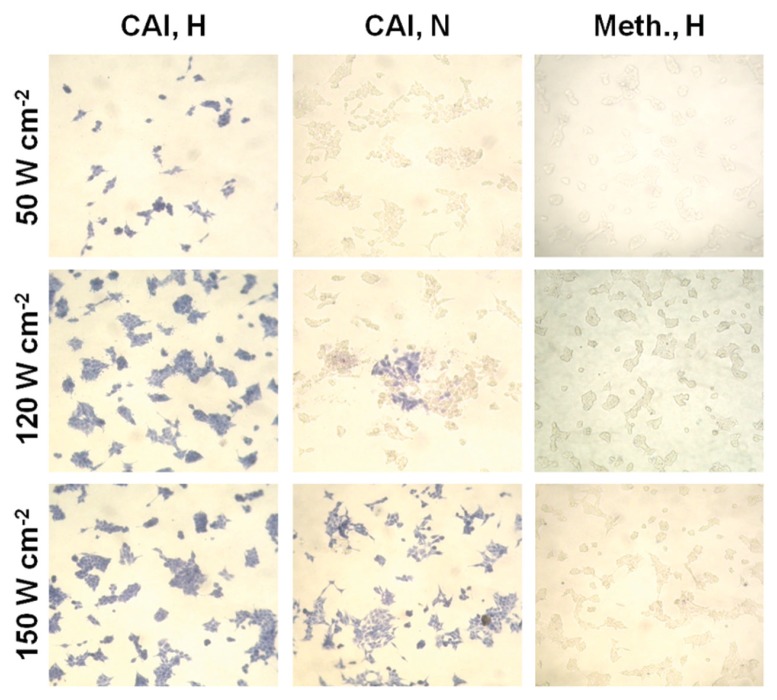
470 μm × 400 μm Optical micrographs of HCT cells treated with 100 μM Au particles **16**, **17** with different terminations and under hypoxia (H) or normoxia (N), and then excited with light at 810 nm and different power density for 5 min. After fixation, cells were stained with trypan blue [21].

The SPR-induced effects of the synthesized coated nanorods on HTC cell cultures was assessed both under normoxic and hypoxic conditions. The SPR was induced by using a NIR radiation at 810 nm with a 5 min exposure time and power densities comprised between 50 and 150 W/cm^2^ (Figure 4).

As reported in Figure 4, the HTC hypoxic cells incubated with CAI-conjugated nanorods **16** were killed using a laser with a power density of 50 W/cm^2^. The scale-up of the radiation densities determined more extensive cellular damages. Hypoxic cells treated with 2-methoxyethylamine-modified particles **17** remained viable up to 150 W/cm^2^. Cell death was also observed when normoxic conditions were considered. In this case, the cultures reported damages only when a radiation density threshold of 120 W/cm^2^ was reached. Such an effect was properly ascribed to the residual nonspecific uptake of CAI-coated nanoparticles **16**, as seen by spectrophotometry (results not reported). This study was the first to report and successfully proof the biomedical application of CAI coated nanorods as potential tools for the treatment of hypoxic tumors and therefore expressing the hypoxia-induced hCA IX isoform. Further studies using the in vivo model of the disease will be necessary in order to give full credit to the research outcomes.

## 3. Poly(Amidoamine) (PAMAM) Dendrimers

Dendrimers are defined as repetitively branched molecules that assume spherical and symmetrical morphology. Dendrimeric scaffolds have been found useful for various applications such as the biomedical ones, which are pertinent for the purposes of this review [22]. Some of us were the first to report CAI functionalized poly(amidoamine) (PAMAM) dendrimers as potential drug delivery systems for the management of ocular hypertension in glaucomatous patients [23,24]. The same authors explored any multivalent effect on the CAs from pathogens (i.e., *Vibrio cholerae, Trypanosoma cruzi, Leishmania donovani chagasi, Porphyromonas gingivalis, Cryptococcus neoformans, Candida glabrata, and Plasmodium falciparum*) which was detected only for some of them [25]. The choice of PAMAM scaffolds was mainly based on: (i) demonstrated bioadhesive features on the corneal surface [26]; (ii) the branched tree-like concentric layers, properly referred to as “generations-G_n_”, allow a precise number of functional groups to be incorporated in the macromolecule by means of standard protocols.

**Scheme 4:**
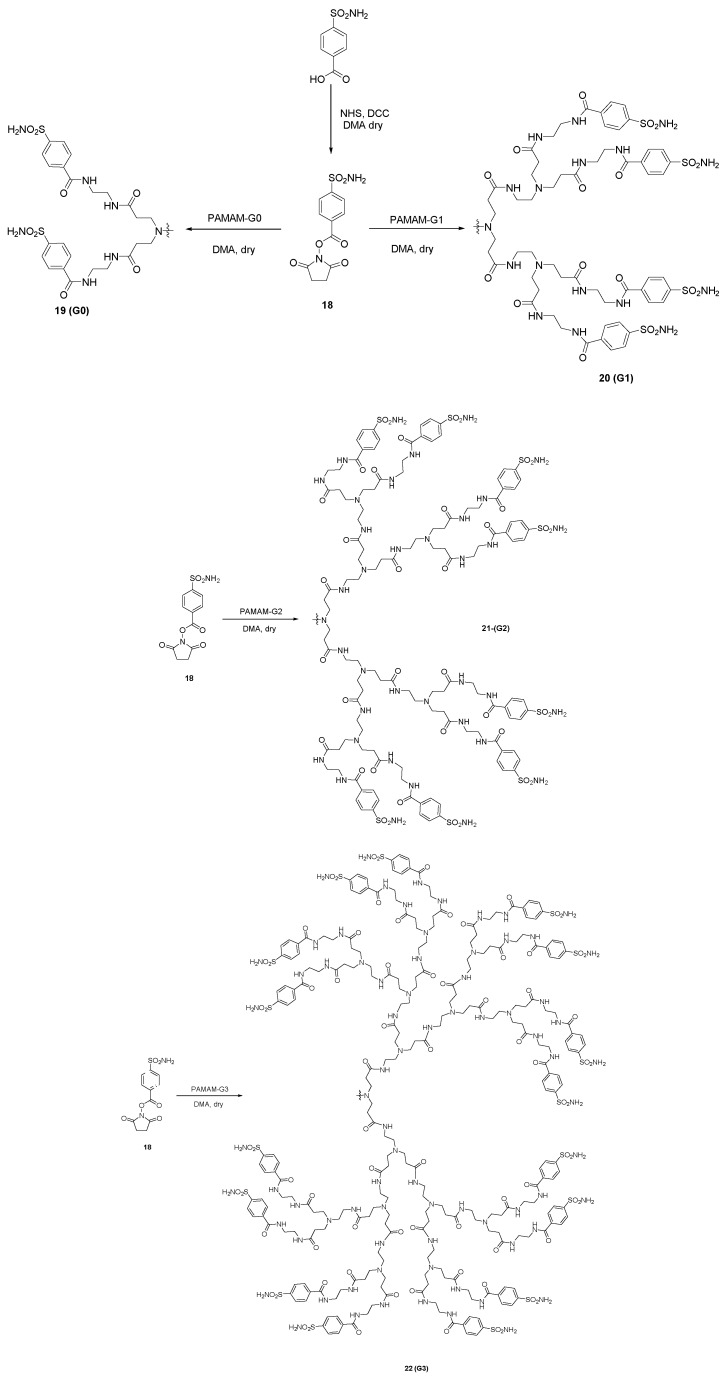
Synthesis of the activated ester **18** and functionalized dendrimers **19**–**22** [21].

The synthetic chemistry approach applied is depicted in Scheme 4 and is based on amide coupling reactions between the G0–G3 dendrimeric terminal primary amines with the freshly prepared *N*-hydroxysuccinimide (NHS)-activated carboxylic ester of the phenyl sulfanilamide **18**.

The activated ester **18**, the sulfonamide functionalized dendrimers **19**–**22** and the reference CAI dorzolamide (**DRZ**) were assayed in vitro for their inhibition properties against the physiologically relevant hCA isoforms (i.e., I, II, IX, and XII) and the data are reported in Table 2 [23].

Overall, the obtained kinetic data showed the dendrimeric derivatives **19**–**22** possess inhibition potencies towards the hCAs tested, which progressively increase with the generations (Table 2). Such a trend was particularly evident for the hCA isoforms II and XII as the G2, and G3 dendrimeric generations reached sub-nanomolar inhibition values (Table 2). As reported in Table 2, effects of multivalence were clearly observed for the four generations of CAI coated dendrimers explored in comparison with the monovalent CAI **1** (Table 1). Such an effect was particularly evident in the case of hCA II and XII, where the decrease of the inhibition constants (*K*_I_s) from G0 to G3 registered potency improvements (*rp*) of 9142 for hCA II and of 6333 for hCA XII respectively. The *rp* normalized data onto the sulfonamide units (*n*) inserted in each dendrimer (*rp*/*n*) were of 285.7 for hCA II and of 197.9 for hCA XII, respectively, thus indicating significant multivalency effects.

The same study reported the successful application of such compounds for the management of ocular hypertension in an animal model of glaucoma. Quite interestingly, all G0–G3 CAI coated dendrimers showed intraocular pressure (IOP) lowering effects far more superior and time lasting when compared to the standard CAI **DRZ** all used at the same concentration (i.e., 2%). In particular, the CAI derivatized G1–G3 generations showed a remarkable ability to maintain constantly reduced IOP values over the experiment time course of 120 h. Although additional studies were not conducted, it is reasonable to speculate that such performances were obtained by means of various effects collectively taking place, which among others, include the release of the CAI units by hydrolytic processed [26] and trans-corneal penetration of the CAI-dendrimers.

## 4. Other Platforms (Polyols, Fullerene, Cyclopeptide)

**Figure 5:**
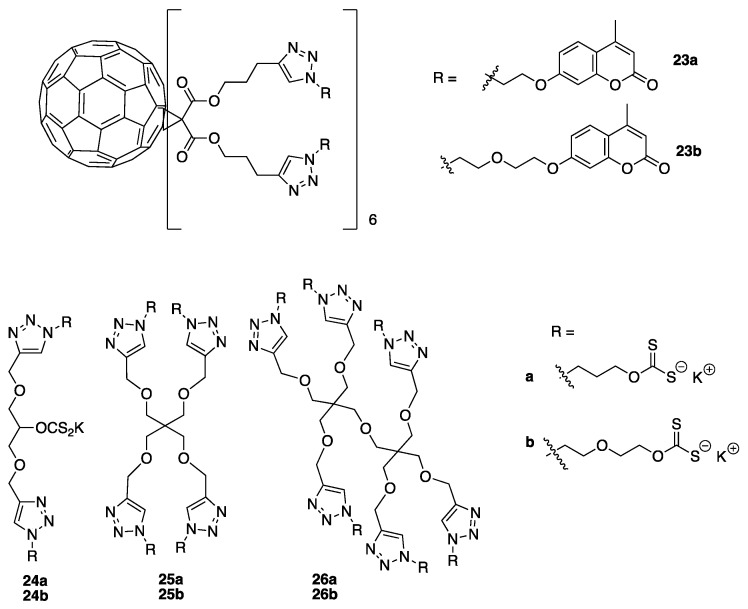
Multimeric systems described by Vincent et al. [27,28].

Research works have also been described with other multivalent platforms bearing different types of CA inhibitor chemotypes. Xanthate and coumarin chemotypes were, respectively, described as multimeric CAIs by Vincent group using respectively fullerene platforms **23** and polyol-scaffolds **24**–**26** (Figure 5) [27,28]. These multivalent systems were elaborated using the click-type copper-mediated azide-alkyne cycloaddition (CuAAC) reaction, as shown in Figure 5. The multivalent systems were in each case, better inhibitor than the monovalent inhibitor, but the weak multivalent effect was observed. However, both studies showed that the multimeric presentation of the CAIs can influence the selectivity against the different isoforms (cytosolic vs. membrane bounds). In the case of polyol-scaffold bearing xanthate chemotypes, weak inhibitory activity (micromolar range) and weak multivalent effects were observed. Nevertheless, improved potency and selectivity were noticed compared with the monovalent analogs [27,28]. The most important results in this work were the demonstration that inactive monovalent inhibitors on hCA II and hCA IX (inhibition constant >100 μmol) can become active when they are ligated on a multimeric platform, with inhibitory activity ranging from 10 to 70 μmol.

**Figure 6:**
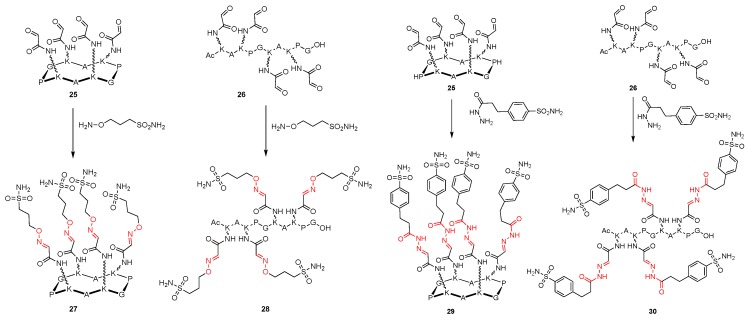
Multivalent CA inhibitors from functionalized peptide scaffolds with oxime or hydrazide ligation (in red). [30].

In 2017, our group reported new multimeric CAIs using functionalized peptide scaffolds as a multivalent platform [29]. We constructed cyclic and linear peptides bearing multiple reactive glyoxylic aldehyde functions able to be conjugated chemoselectively with sulfonamide inhibitors under mild conditions and in high yields using metal-free click-type bioconjugation methodologies such as hydrazone and oxime ligations (Figure 6).

Both peptide scaffolds **25** and **26** were synthesized using automated solid-phase peptide synthesis (SPPS). To prepare the cyclic peptide, cyclization under high-dilution conditions was followed by oxidative cleavage of serine side-chains in order to unmask the glyoxylic aldehyde groups. The synthesis of the linear peptide was realized via acetylation of the N-terminal position, followed by cleavage and deprotection, and finally, oxidative cleavage of serine side-chains. Hydrazone and oxime ligation were performed respectively with the corresponding monovalent hydrazide or oxyamine sulfonamide inhibitors. [29]

The inhibitory activity showed that multivalent oxime conjugates **27** and **28** were less potent than the monovalent system against the membrane-bound isoforms hCA IX and hCA XII. The only improvement of potency was observed against hCA II with a relative potency (*rp*) increased by 13- and 5-fold, respectively.

Multivalent hydrazide conjugates **29** and **30** showed an improved potency against hCA II, hCA IV, and hCA XII compared to the monovalent inhibitors. Moderate multivalent effects against isoforms II, IV, and XII with *rp*/*n* > 1 were characterized. In each case, the pre-organized multivalent conjugates made of the cyclic scaffold, **27** and **29**, were more potent than the conjugate made of the linear scaffold. The results obtained in this study suggest that the multivalent effects act through an increase in the local concentration of CA inhibitors against the enzymes. [30]

## 5. Conclusions and Prospects

The multivalency approach was successfully applied to the design of CA inhibitors belonging to a variety of classes (sulfonamides, dithiocarbamates, carboxylates, etc.) [29,31,32] and applying various multimeric systems, such as gold nanoparticles, gold nanorods, silica nanoparticles, dendrimers, fullerenes, polyols, peptides, etc. In most cases, interesting multimeric effects were observed for these inhibitors compared to the corresponding “monomeric” CA inhibitor from which they were derived. However, in many other cases, the differences were relatively small. This may probably be due to some of the constraints that were imposed on the scaffolds of the CAIs used in the syntheses, and/or to the various platforms employed for derivatization. Thus, although the chemistry of the CAI allows for a huge variety of CAIs and CA inhibition mechanisms, the platforms used to date are rather limited, with few NPs, just one example of dendrimer and quite a few other investigated such scaffolds. Thus, more intense research on the derivatization of novel platforms, with improved (or diverse) properties is to be expected in the future. Furthermore, with few exceptions, the sulfonamides were the most investigated chemotype as CAI warhead. However, many other much more interesting chemotypes are available nowadays [29,31,32], which might lead to multivalent CAIs with improved desired properties. We expect in the future years a large increment in this part of our knowledge of CAIs.

## Figures and Tables

**Table 1:** CA inhibition data against intermediates **3a** and **3b** coated nanoparticles **4a** and **4b** and Au shell against the hCAs I, II, and IX in comparison with the standard CAI **AAZ**. Data in parentheses refer to the *K*_I_ values after 2 h of incubation [11].

Compound	*K*_I_ (nM) *		
	hCA I	hCA II	hCA IX
**3a**	214 ± 9	230 ± 10	41 ± 2
**3b**	>50000	50000	>50000
**4a**	581 ± 18 (128)	451 ± 21 (116)	32 ± 2 (34)
**4b**	28550	30400	31050
**Au**	32000	31600	29560
**AAZ**	250 (± 12)	12 (± 1)	25 (± 1)

* Mean from three different assays, by a stopped-flow technique (errors were in the range of ± 5–10 % of the reported values).

**Table 2:** CA inhibition data against isoforms hCA I, II, IX, and XII with compounds **19**–**22** and **AAZ** and **DRZ** as standards, by a stopped-flow CO_2_ hydrase assay.

Compound	*K*_I_ (nM) *			
	hCA I	hCA II	hCA IX	hCA XII
**18**	7800	640	475	380
**19 (G0)**	24.1	10.4	34.7	9.3
*rp*	323	61	13	40
*rp*/*n*	80.9	15.3	3.4	10.2
**20 (G1)**	12.0	3.1	20.5	1.1
*rp*	650	206	23	345
*rp*/*n*	81.2	25.8	2.8	43.1
**21 (G2)**	10.8	0.93	8.6	0.94
*rp*	722	688	55	404
*rp*/*n*	45.13	43	3.5	25.3
**22 (G3)**	10.5	0.07	5.1	0.06
*rp*	742	9142	93	6333
*rp*/*n*	23.21	285.7	2.9	197.9
**AAZ**	250	12	25	5.7
**DRZ**	50000	9	52	3.5

* Errors in the range of ± 5% of the reported values, from three different assays. *rp*: relative potency = *K*_I_(18)/*K*_I_(Gn). *rp*/*n*: relative potency/number of sulfonamide units.

## Abbreviations

hCA	Human carbonic anhydrase
CAI	Carbonic anhydrase inhibitor
